# Genetic and morphological identification of filarial worm from Iberian hare in Portugal

**DOI:** 10.1038/s41598-022-13354-3

**Published:** 2022-06-03

**Authors:** F. A. Abade dos Santos, M. D. Duarte, C. L. Carvalho, M. Monteiro, P. Carvalho, P. Mendonça, P. C. L. G. Valente, H. Sheikhnejad, H. Waap, J. Gomes

**Affiliations:** 1 Centre for Interdisciplinary Research in Animal Health (CIISA), Faculdade de Medicina Veterinária, Universidade de Lisboa, Avenida da Universidade Técnica, 1300-477 Lisboa, Portugal; 2 Instituto Nacional de Investigação Agrária E Veterinária (INIAV, I.P.), Quinta Do Marquês, Av. da República, 2780-157 Oeiras, Portugal; 3 Associate Laboratory for Animal and Veterinary Sciences (AL4AnimalS), Vila Real, Portugal; 4 InnovPlantProtect Collaborative Laboratory, Department of Protection of Specific Crops, 7350-478 Elvas, Portugal

**Keywords:** Microbiology, Molecular biology, Zoology

## Abstract

The Iberian hare (*Lepus granatensis*) is an endemic species of the Iberian Peninsula and the only hare species found in Portugal, although also being present in some areas of Spain. The reduction of wild hare populations due to several ecological and sanitary factors, has been raising growing concerns in the recent years. Despite different helminth species were already described in Iberian hares in Portugal, to this date, no filarial worms have been identified in this species. Furthermore, only a few studies on lagomorphs’ onchocercid worms are available, referring to other hosts species of hares and/or rabbits. In this study, we describe the presence of filarial worms in the blood vessels of two adult Iberian hares collected in 2019 in continental Portugal. Morphology and sequencing data from the 12S *rRNA*, *coxI*, 18S *rRNA*, *myoHC*, *hsp70* and *rbp1* genes, showed that the filaroid species were genetically related with *Micipsella numidica*. However, the extension of the genetic differences found with *M. numidica* suggests that the filaroids specimens under study belong to a new species, that we provisionally named *Micipsella iberica n. sp.*. The body location of this putative new parasite species and its physiological implications indicate that it may constitute a potential menace to the already fragile Iberian hare justifying, therefore, further investigation regarding the morphological characterization, prevalence and real clinical impact of this new parasite in hares.

## Introduction

Some of the most relevant human infectious diseases are vector-borne parasitosis such as the filarial worms responsible for lymphatic filariasis, like *Wuchereria bancrofti* Wilson, 1822 and *Brugia malayi* Brug, 1927. Many vector-borne parasites are also responsible for disease in domestic and wild animals. Filariods (superfamily Filarioidea) are nematode parasites that can be found in different organs of the definitive host, with a biological cycle characterized by the release of microfilariae into the blood vessels, which are ingested and transmitted to another primary host by an intermediate hematophagous host, an arthropod. Filaroids are frequently reported in Europe where some species are endemic^1^. These species have different pathogenicity for man and animals, representing a growing concern^1–3^.

While, for example, *Dirofilaria immitis* threatens dogs and cats, causing a severe and often fatal cardiocirculatory disease referred to as ‘heartworm disease’, the zoonotic Dirofilaria repens Railliet & Henry, 1911 induces a non-pathogenic subcutaneous or connective muscular fasciae infestation in dogs but is more frequently found in man^4^.

Until now only a few species of filarial worms were described in hares, namely from *Dirofilaria* and *Micipsella* genera. The *Micipsella* genus includes three different species, namely *M. numidica* Seurat, 1917, first described in rodents^5^ but also reported in 2016 in the European hare (*Lepus europaeus*)^6^, *M. indica* Rao, 1938 described in the Indian hare (*Lepus nigricollis*) on India^7^, and *M. brevicaudata* Lyons et Hansen, 1961 described in the American desert hare (*Lepus californicus*)^8^. A revision of the most relevant features of all Onchocercidae described so far on lagomorphs, including several hare species and cottontail rabbits, is summarised in Table 1. Presently, there are no molecular studies in the Iberian Peninsula that have unequivocally identified the species of parasites that have been found. In onchocercids (member of Superfamily Filariodea, Family Onchocercidae), also known as filarial worms or filarioids, the vulva is posterior to the nerve ring, contrarily to filariids (members of Family Filariidae), where the vulva is anterior to the nerve ring.

**Table 1:** Information on relevant filarial species found in rabbits and hares.

Subfamily	Species		Geographic distribution (Year of 1st description)	Host		Location in the host	Morphological particularities		Observations	Occurency reports
							Male	Female		
Splendidofilariinae	*Micipsella numidica* Seirat, 1917		Central and Oriental Asia (1954) Equatorial Africa (1911) Europe (1956)	*L. habessinicus* *L. chadensis* *L. aegyptiacus* *L. europeus* *L. capensis*		Peritoneal cavity Intestinal mesentery	Length: 55–76 mm; Width: 410–736 µm; Presence of caudal cuticular protuberances. Long-tail; 2 to 3 pairs of postanal papillae; Length of right spicule < 100 µm	Length: 47–145 mm; Width: 540–750 µm; Microfilaria without sheath	Absence of *Wolbachia* endosymbiont	^6,18,19^
	*Micipsella brevicaudata* Lyons et Hansen, 1961		North America (1961)	*L. californicus*		Peritoneal cavity	Dimensions unknown; Short and straight tail without post-cloacal papillae and small cuticular bumps; Compared with *M. numidica*, *M. brevicaudata* is less width; Nerve ring is closer to the anterior end;	Microfilariae with sheath		^8^
	*Micipsella indica* Rao, 1938		India (1823)	*L. nigricollis*		Circulatory system (heart and portal vein)	Length 70–100 mm; Long tail, 6 to 7 pairs of pre-anal papillae (three pairs of post-cloacal papillae); Right spicule longer than 110 mm, e.g., longer than those of *M. numidica* and *M.brevicaudata* Small caudal cuticular bumps are absent; Large cut	Length: 120–140 mm		^6,7^
	*M. iberica* n. sp.		Portugal (2019)	*L. granatensis*		Circulatory system (thoracic veins and portal vein)				This study
Dirofilariinae	*Loaina scapicep* Leidy, 1886s*/* *Dirofilaria scapiceps???*		North America, (1984) England (1958)	*L. americanus* *L. campestris* *L. washingtonii* *L. europaeus* *Sylvilagus floridanus* *S. palustris* *S. aquaticus*		Subcutaneous (lumbar region). Intermuscular fasciae of the hock of the foot, and in the joint between the tibia, fibula and tarsal bones. Rarely in the neck and knee joint In *S. floridanus*, worms were free within the delicate connective tissue sheaths around tendons along the front and lateral surfaces of the distal third of the tibiofibular, and immediately above the joint capsule of the ankle In *L. americanus*, worms were found within capsules around tendons along the front and lateral surfaces of the distal third of the tibiofibular, and immediately above the joint capsule of the ankle	Length: 11–16 mm; Width: 305–375 µm; Left spicule width 113–139 µm; Right spicule length 84–86 µm; The distal end barbed Ends tapered; Lateral alae; Additional pair of ventral, sub terminal papillae near the tip of the tail	Length: 25–30 mm; Width: 745–765-µm; Vulva located around 1,5 mm from the anterior end; Embryos are slender and filiform Tapered ends; *L. scapiceps* is coiled helically along the entire body length	*Aedes canadensis*, *A. cinereus*, *A. excursians*, *A. fitchii*, and *A. verlans* are putative intermediate hosts	^20–22^
	*Loaina uniformis* Priece 1957 *Dirofilaria uniformis*		USA (1957)	*Sylvilagus floridanus*		Subcutaneous tissues of the trunk	Body not coiled; Ends are not significantly tapered; No lateral alae; The distal end is smooth and pointed			^23,24^
	*Dirofilaria timidi*		Russia (1966)	*Lepus timidus*		Thoracic cavity	Considered as a species inquirenda			^25,26^
Onchocercinae	*Cercopithifilaria leporinus* Bartlett,1983		Canada (1983)	*Lepus americanus*		Subcutaneous connective tissues of trunk	Length: 7.1–10.3 mm; Width 94-114 mm; The pattern of papillae on the male tail Posterior end of body spirally coiled in 1 to 2 turns Area rugosa present, consisting of transverse bands of small, longitudinally elongate bosses, commencing 1.53 mm anterior to anus; Perianal, postanal, and subterminal caudal papillae present Perianal group: single, mid-ventral, sessile papilla immediately anterior to anus; 2- 3 small, sessile to semi-pedunculate papillae immediately posterior to anus; 5–6 medium sized pedunculate papillae lateral or slightly posterior to anus Postanal group: 1–2 large, pedunculate papillae located subventrally, one on either side, approximately midway between anus and posterior extremity Subterminal group: 3–5 variably sized occasionally double, semi-pedunculate papillae in two subventral Caudal extremity terminating in cuticular cone and two cuticular petaloid appendages Narrow caudal alae present Spicules dissimilar and unequal Proximal portion of lamina complex and twisted, distal portion simple and rod-like Right spicule 94 µm long, simple, non-granular in appearance, and strongly sclerotized except for right distal half which is weakly sclerotized. Gubernaculum absent	Length: 12.2-25 mm. Width: 104–180 mm Small size numerous mucrons on the tail; Vulva leading into a large, spherical vestibule One postdeirid occasionally present in left or right lateral field, approximately midway between anus and posterior extremity; Single, lateral papilla occasionally present posterior to postdeirid One to two lateral, subterminal papillae occasionally present; One to three lateral, postdeirid-like, subterminal structures occasionally present Posterior extremity complex, with terminal cuticular cone and two cuticular petaloid appendages Numerous, variably sized, cuticular mucrons located subterminally, ventral and dorsal to terminal cone		^26^
	*Brugia lepori* Eberhard, 1984 *Brugia* sp.		North America (1994)	*S. floridanus* *S.aquatics*		Abdominal lymphatics and subcutaneous tissue	Length: 294–344 µm; Width: 8–8.5 µm (Microfilariae) Microfilaria had the characteristic subterminal and terminal nuclei		*Brugia* sp. microfilariae were observed in more than 60% of wild rabbits collected on Nantucket Island, Massachusett. *B. leporis* is a putative source of human infections in North America	^27,28^

Although fundamental, the classic morphology-based identification has proved insufficient for nematode identification, as well as to understand the existent phenotypic diversity, mainly due to the sparse morphological variations found among closely related taxa. Given the high degree of morphological intraspecific variability and the interspecific similarities, taxonomical classification is sometimes extremely complicated and controversial. For instance, regarding genus *Lernaea,* morphology is not a reliable taxonomic tool as molecular data and experimental infection reveal that *L. cyprinacea* Linnaeus, 1758 and *L. cruciata* Le Sueur, 1824 are conspecific^9^.

Different molecular methods, ranging from fingerprint to sequencing analyses, but also protein-based information, have been used to complement morphology-based data and circumvent their limitations. Reclassification of organisms is often based on genetic data, rather than on the phenotype or other biological features^10^. Partial sequences of mitochondrial and nuclear genes have been largely used to classify species of eukaryotic parasites and revolutionized our understanding of the distribution and evolutionary history of several parasites occurring worldwide^11^. In these studies, partial sequences of mitochondrial ((12S rRNA and *coxI* (cytochrome oxidase subunit I)) and nuclear genes (*18S rDNA* (that encodes the 18S rRNA), *myoHC* (that encodes the myosin heavy chain), *hsp70* (that encodes the 70 kilodalton heat shock protein), *rbp1*(that encodes the RNA polymerase II large subunit)), were used for species identification and comparison^12–14^. Furthermore, the combined analyses of nuclear and mitochondrial markers have proved successful in species discrimination or to explore available molecular data^15^. Recent studies have described new parasite species by concatenating several genes sequences, in order to increase the discriminatory power of the phylogenetic analyses when comparing onchocercid species^13,16,17^.

In this study, we report the presence of filarial parasites in two adult Iberian hares, carrying out an in-depth investigation regarding their taxonomic classification. The results support the occurrence of a new species of *Micipsella* in Iberian hares. The hares in which the filarial worms were observed were received by the National Reference Laboratory for Animal Diseases, within the scope of a wild leporid populations health status assessment carried out in Portugal between August 2017 and June 2020 by the Project +Coelho (Dispatch 4757/17, 31th may). During this period, dozens of dead Iberian hares were submitted to necropsy and pathological examination.

## Materials and methods

### Sample origin

The animals used in this study were collected dead from the field. No animals were handled or euthanized for the purposes of this study.

An adult male Iberian hare (hare-1, 25456PT19) with a poor body condition (1.7 kg), was collected in Beja district, Portugal, on the 22nd of August of 2019. A second specimen, an adult male (hare-2, 35468PT19) with a good body condition (2.2 kg) was collected in Portalegre district, Portugal, on the 10th of November 2019. While the first animal arrived fresh at the Instituto Nacional de Investigação Agrária e Veterinária (INIAV, I.P.), the second was received frozen. Several organ samples from both hares collected during the necropsy were fixated in 10% neutral buffered formalin (w/v), routinely paraffin-embedded, sectioned at 4 µm, and stained with Hematoxylin and Eosin (H&E). During necropsy, three filarial parasites were observed in hare-1 and four parasites in hare-2. Those specimens were collected and preserved in 70% ethanol for morphological identification.

### Morphological characterization

Morphometric analysis was based on one adult filaria from hare-1, and three adults from hare-2. The length of the filariae was measured in a plastic tray before a small middle section was cut for molecular analysis. For microscopic visualization, the anterior and posterior parts of the parasites were mounted on glass slides using lactophenol, for clarification. All observations and measurements were carried out on a Leica DM IL LED Inverted Microscope (Leica, Wetzlar, Germany) and photographed using a LEICA EC3 photography system (Leica, Wetzlar, Germany). Macroscopic drawings were performed manually, using an Olympus BX51 microscope with an Olympus™ Drawing Attachment Tube (Olympus, Hamburg, Germany).

### DNA extraction

For nucleic acid extraction, 5 mm fragments of the middle section of six parasites were incubated with 20 μL proteinase K (600 mAU/mL) in 200 μl PBS (w/v) and submitted to extraction using the MagAttract 96 cador Pathogen Kit (Qiagen, Hilden, Germany) in a BioSprint 96 nucleic acid extractor (Qiagen, Hilden, Germany), according to the manufacturer’s protocol. DNA concentration was determined by A260 measurement (Qubit 4 Fluorimeter by Invitrogen, California, USA). Nucleic acids were preserved at − 20 °C until use.

### Molecular characterization

Polymerase chain reactions (PCR) targeting mitochondrial and nuclear genes were performed in a final volume of 25 μl under the following final conditions: 1x buffer including 1.5 mM MgCl_2_, 0.2 mM of each deoxynucleotide triphosphate (dNTP), 1 mM each of forward and reverse primers, 1 unit of the DNA polymerase (High Fidelity DNA Polymerase, Roche, Basel, Switzerland) and 20 ng of genomic DNA. To assess the specificity of the reactions, DNA extracted from *Dirofilaria immitis* Leidy, 1856, was included in each PCR, as a positive control. Several pairs of primers were used to amplify the partial sequences of two mitochondrial genes (12S rDNA (~450 bp) and *coxI* (~600 bp)), and four nuclear genes (18S rDNA (~740 bp), *myoHC* (~785 bp), *rbp1* (~ 640 bp) and *hsp70* (~610 bp)), as described by Lefoulon et al.^13^.

Amplicons from two parasites (one from each hare) were purified using the NZYGelpure kit (NzyTech, Lisbon, Portugal) following the manufacturer’s instructions and then directly sequenced using the ABI Prism BigDye Terminator v3.1 Cycle sequencing kit on a 3130 Genetic Analyser (Applied Biosystems, Foster City, CA, U.S.A). The obtained nucleotide sequences (Table 2) were analysed and assembled into consensus sequences using the BioEdit version 7.2.5 software and submitted to GenBank (Genbank access numbers are provided as Supplementary data, Table S1). Nucleotide sequences were translated using MegaX10.1 software.

**Table 2:** Percentage of nucleotide similarity between *M. iberica* genes obtained from hare-1 and hare-2 and other filarioid genes sequences from public databases.

Gene (sequence size) Acession numbers	Differences between Filariae from hare-1 vs hare-2	Percentage of similarity (BLASTN on 15.04.2021)
*12S rDNA* (486 nt) MW928503 MW928504	Addition of 2 nt (TT)	90.06% with *M. numidica* (KR091069.1) 88.25% with *Chandlerella quiscali* Linstow, 1904 (KX768276)
*coxI* (649 nt) MW934617 MW934618	None	91.35% with *M. numidica* (KR232089) 89.78% with *D. repens* (MH780817)
*18S rDNA* (590 nt) MW928501 MW928502	None	99.15% with *Rumenfilaria andersoni* (KP760163.1) 99.15% with unidentified filarial species from Finnish cervids (EF081340.1)
myoHC* (661 nt) MW928499 MW928500	None	96.97% with *Rumenfilaria andersoni* (KP760309)
hsp70* (631 nt) MW928505 MW928506	None	92.52% with *Rumenfilaria andersoni* (KP760456) 84.16% with *Madathamugadia* hiepei Hering-Hagenbeck, Boomker, Petit, Killick-Kendrick & Bain, 2000 (KP760242)
rbp1* (561 nt) MW934615 MW934616	None	93.89% with *Madathamugadia hiepei* (KP760293) 92.52% with *Rumenfilaria andersoni* (KP760456)

*Micipsella sequences not available.

### Phylogenetic analysis

The partial sequences of each gene were aligned separately using Mega X software and manually assembled. For phylogenetic analysis based on peptide and nucleotide alignments, doubtful residues, and the nucleotides in the same position, were removed, in all sequences. The *12S rDNA*, *18S rDNA*, *coxI*, *myoHC*, *hsp70* and *rbp1* partial nucleotide sequences of the filaroids understudy were concatenated to obtain a more robust phylogenetic analysis, according to Lefoulon et al.^13^. The two concatenated sequences obtained separately from hare-1 and hare-2 filariae, were aligned with the homologous concatemers constructed from sequences available in the database. To allow comparison with previous works, most of the sequences used by Lefoulon et al.^13^, were also included in our phylogenetic analysis.

The phylogenetic trees were obtained in Mega X, by Maximum Likelihood using the General Time Reversible model (GTR), with gamma distribution and/or allowance of invariant sites and 10 Gamma Categories (Gamma with invariant sites option). Evolutionary analyses were conducted in MEGA X. Pairwise identities and the heat map for nucleotide identity matrix were calculated using SDT v1.2^29^.

### Declaration

This study did not use live animals and was carried out within the scope of a National Plan for the Control of Rabbit Haemorrhagic Disease Virus 2 in rabbits (Dispatch no. 4757/2017 of 31 May), with the legal authorisations from the National Authority-the Institute for Nature Conservation and Forests (Instituto da Conservação da Natureza e das Florestas, I.P. ICNF).

## Results

### Necropsy and histopathology

**Figure 1:**
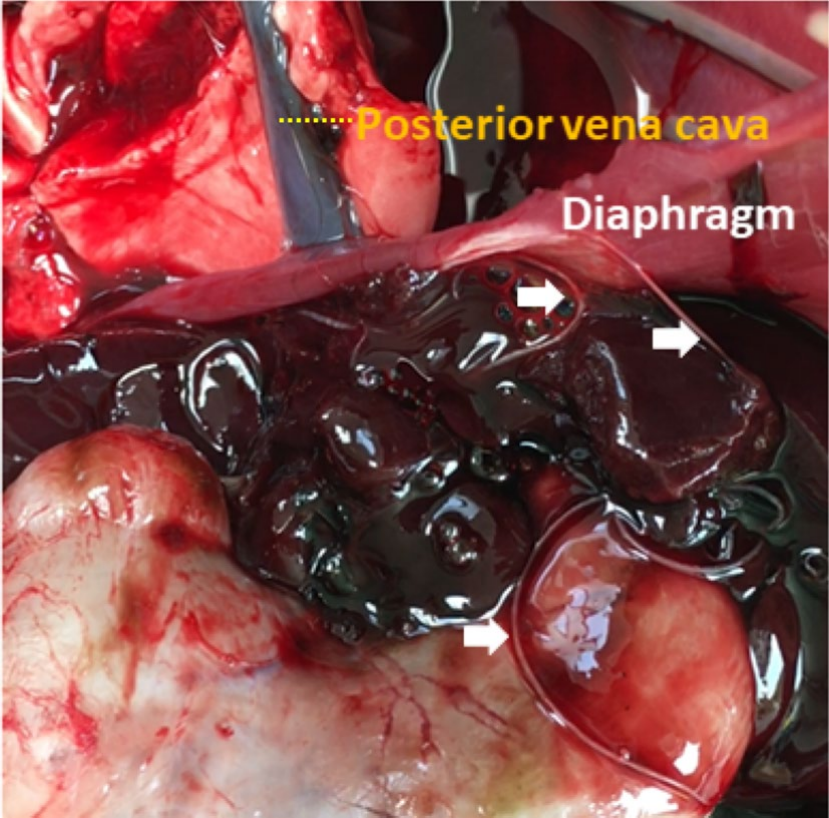
Necropsy from hare-1. Incision of posterior vena cava, in its passage through the diaphragm, exposed several specimens (black arrows) of parasites, still with mobility.

**Figure 2:**
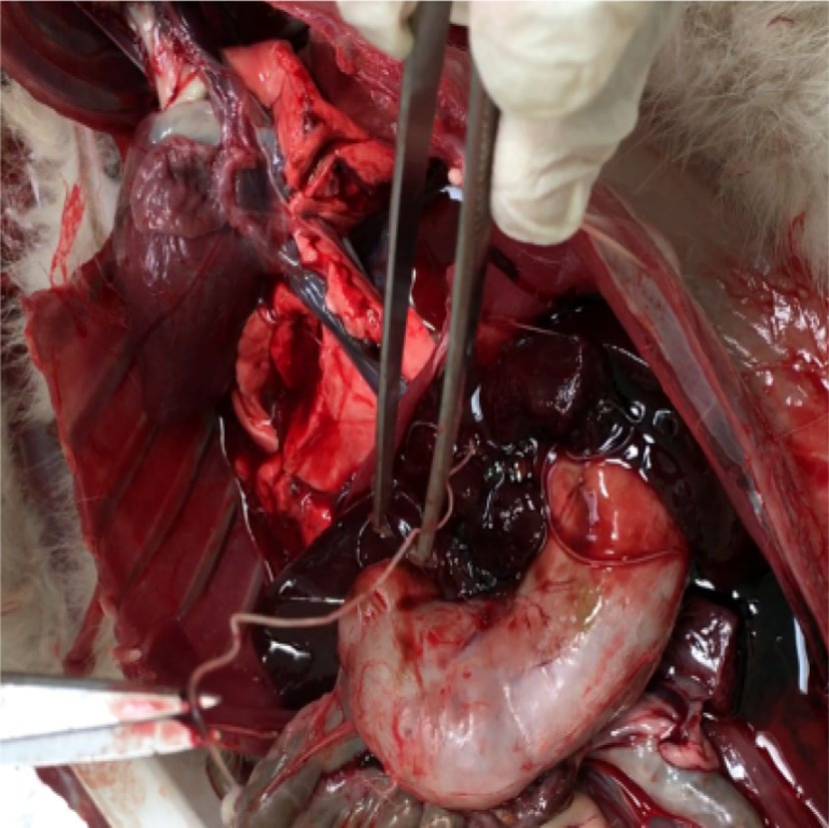
Necropsy from hare-1. One of the filariae observed is held by tweezers and scissors.

**Figure 3:**
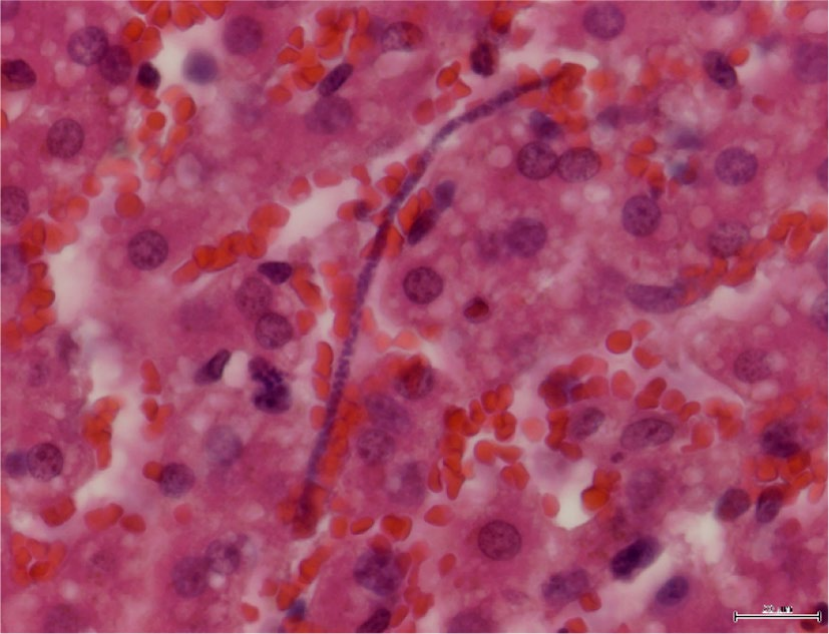
Liver from hare-1. Presence of a microfilaria inside a sinusoid. ×400, H&E.

**Figure 4:**
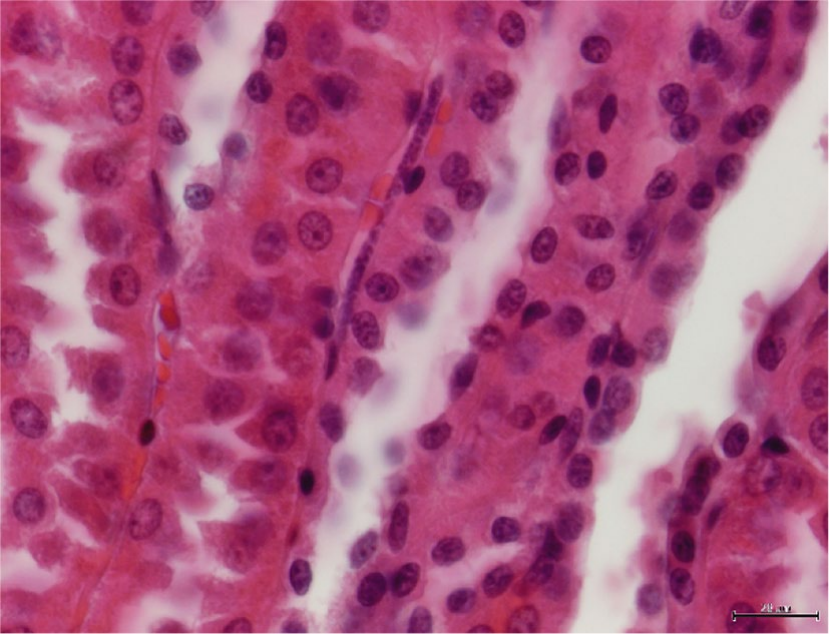
Kidney from hare-1. Presence of a microfilaria in an interstitial tissue vessel. ×400, H&E.

At necropsy, three alive filarial worms were collected from the posterior vena cava of hare-1 and four dead specimens were obtained from the thoracic cavity of hare-2, more specifically from the interior of the vessels next to the diaphragm (Figs. 1 and 2). The worms were preserved in 70% ethanol for morphological and molecular characterization. Severe microfilaremia was found during the histopathological analysis of hare-1, mainly in the liver (Fig. 3), lungs, kidneys (Fig. 4), and spleen, but also in other organs such as the testis.

### Morphological characterization

**Figure 5:**
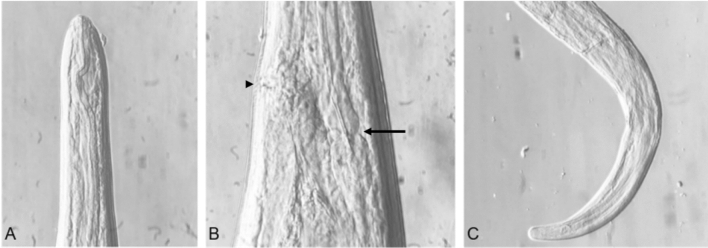
(**A**) Anterior extremity of a female specimen from hare-1 (×100), (**B**) Joint zone of the oesophagus with the intestine (arrow) and vulva (arrow head), female, hare-1 (×200), (**C**) Posterior extremity of a female specimen, hare-1. (×100).

**Figure 6:**
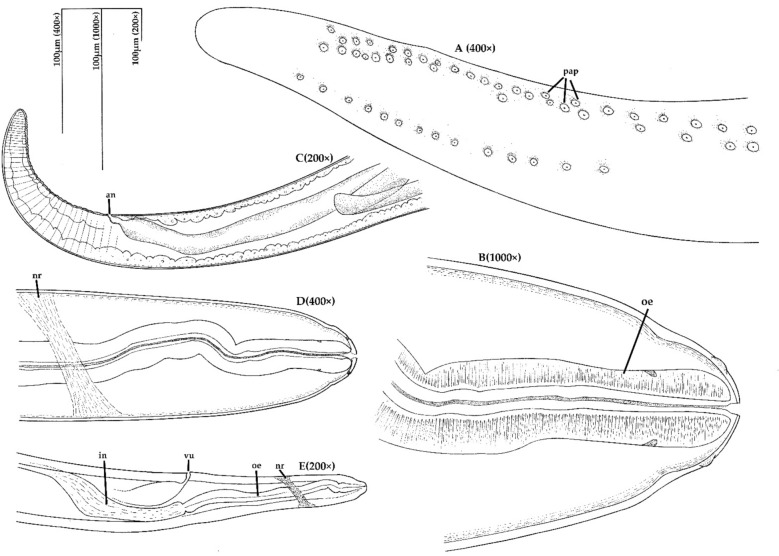
Schematic view of anterior and posterior extremities of a female specimen. (**A**) Posterior part, immature female, ventral view; (**B**) anterior part, ventral view; (**C**) posterior part, lateral view; (**D**) anterior part, ventral view; (**E**) anterior part, lateral view. Abbreviations: an-anus, oe-oesophagus, vu-vulva, nr-nerve ring, in-intestine, pap-papilla.

Two female filariae and one fragmented specimen of undetermined gender were found in hare-1. A total of three female filariae and one fragmented of undetermined gender were observed in hare-2. Regarding the four complete mature specimens (n = 4 females), the mean measurements included a 157 mm (153–165) length, a 652.5 μm (550–780) width, a 740 μm (720–750) oesophagus and distance ranging from the vulva to the anterior extremity of 684 μm (620–800). The nematodes presented a filiform body morphology and tapered ends (Figures 5 and 6). Their extremity was elongated, digitiform, and rounded, and showed a rectilinear oesophagus. In the blood smears, the mean length of the microfilaria was 107.94±3.32 μm.

### Molecular characterization

Amplicons with the expected sizes were obtained by conventional PCR for all genes of the two filariae’ DNA preparations. For all genes investigated, the nucleotide sequences from one filaroid obtained from hare-1 and another from hare-2, showed 100% identity between each other, exception made for the 18S *rRNA* gene, where a 2-nucleotide insertion was observed in the filariae from hare-1, when compared to that from hare-2.

The concatenated sequences for *12S rDNA*, *18S rDNA*, *coxI*, *myoHC*, *hsp70* and *rbp1* genes from hare-1 were used to generate a ML tree (not shown). The identified filarial worms in this study showed a closer phylogenetic relation with *Rumenfilaria andersoni* Lankester & Snider, 1982. The unavailability in public databases of *12S rDNA*, *myoHC*, *hsp70* and *rbp1* sequences for *Micipsella* species prevented us from including these sequences in our phylogenetic analysis. For this reason, a second concatenated alignment, including only the *18S rDNA* and *coxI*, was constructed to compare the filariae sequences from hare-1 and hare-2 with those publicly available from *Micipsella numidica*.

**Figure 7:**
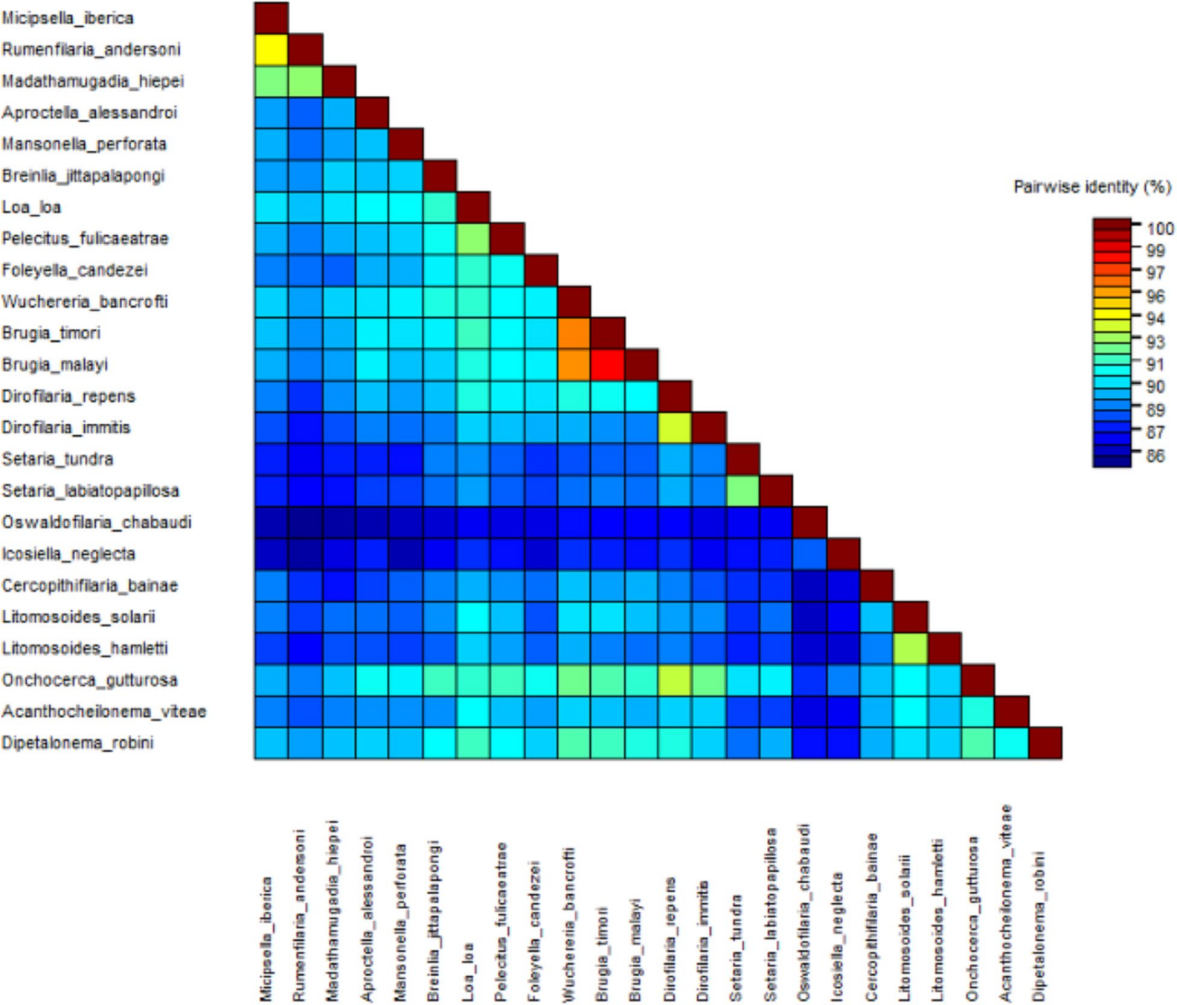
Heat map of nucleotide identity using concatenated partial DNA sequences from *18S rDNA*, *12S rDNA*, *coxI*, *myoHC* and *hsp70* genes.

Figure 7 shows the heat map of pairwise identity using the five gene concatomer. In accordance with the phylogenetic inference, the higher similarity was observed with *Rumenfilaria andersoni* (~94%).

**Figure 8:**
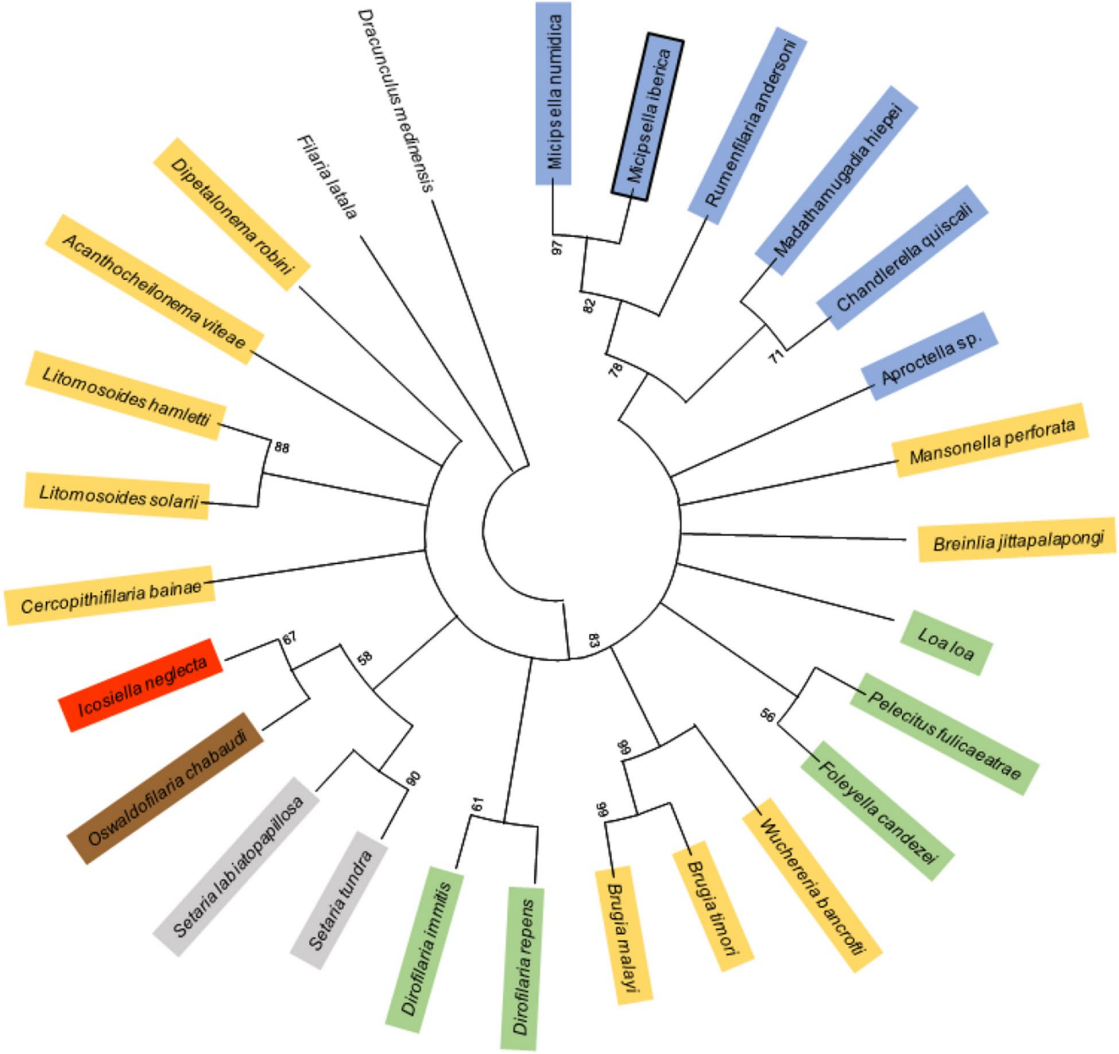
Phylogenetic tree using concatenated partial DNA sequences from 18S rDNA and *coxI*. Ancestral states were inferred by Maximum Likelihood using the General Time Reversible (GTR) model. The tree shows a set of possible nucleotides (states) at each ancestral node based on their inferred likelihood at site 1. This analysis involved 27 nucleotide sequences. There was a total of 777 positions in the final dataset. Evolutionary analyses were conducted in MEGA X. Genbank access numbers are provided as Supplementary data, Table S1.

The phylogenetic analysis using concatenated partial sequences from *coxI* and *12S rDNA* allowed to include a sequence from *M. numidica* (KR232089) previously described in other species of hares namely European hare^6^. The unrooted circular phylogenetic tree is shown in Figure 8.

**Figure 9:**
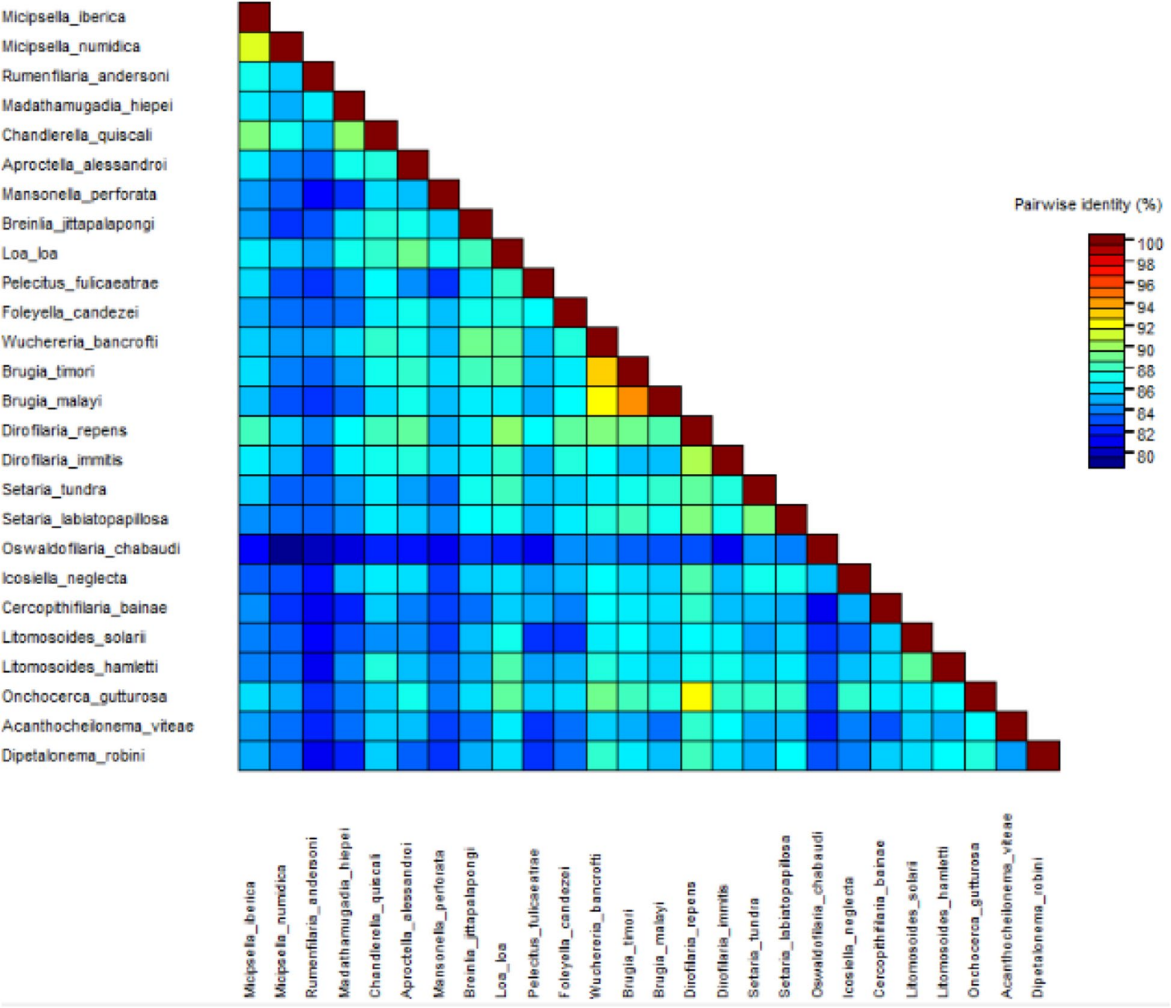
Heat map of nucleotide identity using concatenated partial DNA sequences from *12S rDNA* and *coxI*.

Similarly, the heat map of pairwise identity of *12S rDNA* and *coxI* concatemer using a muscle alignment showed that the studied specimens have the highest similarities (~88–90%) with *M. numidica*, *Chanderella quiscali* Linstow, 1904 and *Dirofilaria repens* Railliet & Henry, 1911 (Figure 9).

## Discussion

In 2019, the International Conservation Union (IUCN) attributed the status of “minor concern” to *Lepus granatensis* in the Red Book of Threatened Species, although a declining trend was recognized^30^. Adding to this continuous reduction, new diseases were recently identified in the species, impacting severely in the survival many local wild populations^30–32^. One of the major parasites threats to Iberian hare is *Cysticercus pisiformis*, the larval stage of *Taenia pisiformis*. Interestingly, during a 35-month survey carried out within the scope of a national surveillance programme in continental Portugal, the incidence of internal parasitism in Iberian hares was lower than in wild rabbits^33^. In this manuscript, we describe the presence of filarial parasites in two adult Iberian hares from South Portugal. Being must less abundant than wild rabbits, the restrictions inherent to the opportunistic sampling of Iberian hares limited the number of filarial worms available for the analysis. Even though no male filarioid specimens were collected from the two hares, the morphological and genetic characteristics based in 12S *rDNA* and *coxI* partial sequences, showed that the filarioid species found resemble *Micipsella* species.

The prevalence of this parasite in the wild populations and its real pathogenic potential is unknown. However, given the adults’ dimensions and their location in the blood vessels, the risk of vascular thrombosis is unavoidable.

Currently, only a few studies are available on onchocercid from hares and rabbits. Known filarioid species from lagomorphs were included in five different genera, namely *Micipsella*, *Loaina*, *Cercopithifilaria*, *Brugia* and *Dirofilaria.*Genus *Micipsella* (Onchocercidae) comprises three recognized species, namely *M. numi*dica (Seurat, 1917), *M. indica* (Rao, 1938) and *M. brevicaudata*^8^. Genus *Loaina*^20^ was created to include *Dirofilaria* species of rabbits from North America and comprises two species, *Loaina scapiceps* (Leidy, 1886) and *Loaina uniformis*^24^, the latter considered as the type species. This genus is morphologically distinct from *Dirofilaria* and other *Dirofilariinae*^20^. *Loaina uniformis* has been reported from subcutaneous tissues of several rabbit species (*Sylvilagus floridanus*, *S. palustris*, *S. aquaticus*) in different USA states. *Loaina scapiceps*, infecting the tarsal bursa of the hind feet, has also been reported in the mentioned above rabbit species as well as in different hare species (*Lepus americanus*, *L. campestris*, *L. washingtonii*). One *Brugia* species has been described on the abdominal lymphatics and subcutaneous tissues of rabbits *(Sylvilagus aquaticus*, *S. floridanus*)^28^. *Brugia leporis*^20^ was reported in rabbits in Louisiana, USA. *Cercopithifilaria leporinus*^26^ was described as a new species from the subcutaneous tissues of the trunk of snowshoe hares (*Lepus americanus*) in Canada^26^. Another species, *Dirofilaria timidi*^25^, was regarded as a species inquirenda. Only partially described, its proper taxonomic group proved difficult to be determined since it does not belong in *Dirofilaria* genus.

*Micipsella numidica* lives in the peritoneal cavity and circulatory system of hares being more rarely found in rabbits. Available information refers that *M. numidica* does not have a very strict location within the host having been found also in the abdominal cavity, between the intestinal mesenteries, in the circulatory system, particularly in the portal vein, the supra-hepatic vein or the capillaries of the greater omentum. Since the portal vein and its direct tributaries are not systematically opened at necropsy, the number of parasitised animals is certainly underestimated and this parasite is probably more widespread than thought. In other hare species, the white-tailed jackrabbits (*Lepus townsendi*) from the USA and the Indian hare (*Lepus nigricollis*), two additional species of *Micipsella* are currently known, namely *M. brevicaudata*^8^, a parasite of the peritoneal cavity and *M. indica*^7^ found in the circulatory system (heart and portal vein), respectively.

*Micipsella numidica* was first described in Algeria in 1917^5^ under the name *Filaria numidica* but in 1921 Seurat created the genus *Micipsella* for this nematode. The first specimens were collected from the abdominal cavity of Desert hares (*Lepus capensis*). Since then, the parasite has been identified several times in different regions of the world, being reported in Armenia^34^, Mongolia^35^, Hungary^18^ and in Italy^6^.

*Micipsella numidica* is a thread-like nematode, tapering at both ends. The tail, in both sexes, is elongated, digitiform and rounded. The anterior end is thinned and forms a hemispherical cap with a mouth at the top, followed by an undivided, rectilinear oesophagus, which is followed by an elongated intestine that widens considerably at its origin. The nerve ring is situated in the anterior third of the oesophagus. The thick cuticle is generally devoid of striation.

Mature female parasites from our study have a length ranging from 153 mm to 165 mm, which are greater than those described by Seurat^5^ (70–140 mm) and Ivashkin (60–93 mm). A similar width and distance of oesophagus from the cephalic end was found between mature females from the three studies (quais?). The vulva opens at 620-800 μm from the cephalic end, similarly to the specimens described by Seurat. Since no male specimens were collected in the current study, no information could be obtained regarding the male morphological distinctive features.

The microfilariae of *M. numidica* measure 95-189 μm long by 3.55-4.4 μm wide. They have no sheath and the posterior end is rounded. *Micipsella indica* is a large Filaria (male, 70 to 100 mm; female, 120 to 140 mm).

The morphology of nematodes is remarkably constrained^36^, exemplified by the challenge that *Caenorhabditis elegans* Maupas, 1900 and *C. briggsae* Briggs, 1944 morphological discrimination pose to most trained nematode taxonomists, besides an estimated date of divergence of 80–110 Mya^37^, long before the segregation between the mouse and human lineages. Since cryptic species must abound in the phylum Nematoda, molecular-based techniques are the only practical approach to recognize and differentiate^36^.

Misidentifications based on morphological approaches resulted in huge economic losses around the world^38^. Nowadays, molecular methods allow the recognition of many new taxa, some based on sequence information alone^39^. Per these authors, the high resolution of sequencing analysis overcomes the limited capacity of morphological, image-based and protein-based methods. Agreeing with this, other authors consider that molecular data is better than morphological data to support phylogenetic studies^40–42^.

The strategy of sequencing different genes and performing a concatenated phylogenetic analysis is being increasingly used worldwide, notably using these aforementioned genes and others such as *myoHC* and *hsp70*, questioning the previously existing classifications based on smaller sequences or on morphology^13^. In this study, we combined mitochondrial DNA barcodes with nuclear data to circumvent the downsides linked to maternal inheritance. For this reason, we used partial sequencing data of set of mitochondrial and nuclear genes to obtain a phylogenetic classification for the two filariae specimens found. The targeted genes included *coxI*, *12S rDNA*, and *18S rDNA*, but also *hsp70*, *myoHC* and *rbp1*.

Cytochrome c oxidase subunit 1 mitochondrial gene (*coxI* or *COI*) is a standardized molecular marker for the comparison and classification of animal species^43^ discriminating vertebrates and invertebrates^44–46^, demonstrating its power as a marker for identification from other closely related animal species. The *coxI* gene appears to provide a better phylogenetic signal than the other mitochondrial genes (e.g. *12S rDNA*, *16S rDNA*)^47,48^. This is thought to be the result from the rapid evolution of *coxI* gene, which allows discriminating between closely related species and to investigate intraspecific diversity^43^. Besides the identification of already known as well as new species, sequencing of *coxI* gene was suggested as a standard for cryptic taxa discovery, an association of different life stages of the same species and wildlife conservation genetics^45^.

The mitochondrial *12S rDNA* is a genetic marker useful to study the molecular systematics of nematodes and to reveal intra-phyla relationships^49^. It is often concatenad to other mitochondrial genes such as *coxI*^13^ and *16S DNA*^49^, to potentiate the discriminatory power of the nucleotide variability.

The small subunit *18S rDNA* gene is the most frequently used marker for taxonomic identification in eukaryotes, phylogeny and evolution investigations^50,51^.

The ribosomal internal transcribed spacer (ITS) region has shown insufficient discriminatory power for nematode classification. However, the nucleotide sequences of a fragment within the small subunit nuclear ribosomal DNA (*18S rDNA* or *SSU*), provides adequate information to identify genera of nematodes and may even distinguish between species^52^.

BLAST analysis of the *12S rDNA* and *coxI* partial sequences of the filariae under study revealed the highest similarities with *M. numidica* (Splendidofilariinae), 90.06% and 91.35%, respectively (Table 2). As refered before, no sequences are presently available for *Micipsella* genus regarding genes *myoHC*, *hsp70* and *rbp1* and *18S rDNA*. Furthermore, given the lower resolution at lower taxonomic levels of nuclear 18S rDNA compared with mitochondrial *12S rDNA*^49^, the *18S rDNA* sequence of the specimens being described showed 99.15% similarity both with *Rumenfilaria andersoni* (KP760163) (Spendidofilariinae) and *Filarioid* sp. (EF081340) (Filarioidea). Also, regarding the partial sequences of *myoHC*, *hsp70* and *rbp1* genes obtained from the filaroids reported here, the higher similarities were observed with *Rumenfilaria andersoni* (96.97%)(KP760258), *Rumenfilaria andersoni* (92.52%)(KP760456) and *Rumenfilaria andersoni* (96.23%)(KP760309), respectively. The genetic distances (Figs. 7 and 9) corroborated the phylogenetic findings.

The pairwise identities based in *coxI* and *12S rDNA* concatenated sequences showed the higher values between species of the same genus, namely ~90% between *Dirofilaria immitis* (Dirofilariinae) and *D. repens*, and 88% between *Brugia malai* (Onchocercinae) and *B. timori*. The similarity between the studied specimens and *M. numidica* of the same order of magnitude (91%), expose the remarkable genetic proximity with *M. numidica* with which they may share the same genus. The *coxI* gene contains a higher level of sequence diversity, particularly in the variable regions, making this region ideal for resolution at lower taxonomic levels^39^.

In conclusion, using molecular data, the parasites were identified as phylogenetically closer to *Micipsella numidica* despite differing from it in 8.65% and 9.94% nucleotide similarity (Table 2). Particularly, the relatively low genetic identity in the *coxI* gene from the two filariods is incompatible with a same species. Along with the morphological differences registered, namely larger females, these findings support that *Micipsella* described in this study and *M. numidica* are not the same species exhibiting morphological intraspecific variability, but instead, two different species. Since the report of *M. numidica* in Iberian hares (Segovia, 2014) was not accompanied by molecular and morphometric data, there is no certainty that the specimens reported at that time in Spain correspond in fact to the species *M. numidica*, and not to the one here described. The possibility that several subspecies may exist within the species *M. numidica*, with the two specimens under analysis being subspecies within this taxon, cannot be further investigated given the scarcity of genetic information available in the public databases to compare with.

## Supplementary Information

Supplementary Information.

## Data Availability

Data are contained within the article. Filaria specimens as well as preserved animal organs are deposited in the National Institute for Agricultural and Veterinary Research biobank and available upon request.

## References

[1] Baneth G (2016). Major parasitic zoonoses associated with dogs and cats in Europe. J. Comp. Pathol..

[2] Morchón R, Carretón E, González-Miguel J, Mellado-Hernández I (2012). Heartworm disease (*Dirofilaria immitis*) and their vectors in Europe - new distribution trends. Front. Physiol..

[3] Otranto D, Dantas-torres F (2013). The prevention of canine leishmaniasis and its impact on public health. Trends Parasitol..

[4] Dantas-Torres F, Otranto D (2013). Dirofilariosis in the Americas: A more virulent *Dirofilaria immitis*?. Parasit. Vectors.

[5] Seurat, L. G. Une nouvelle Filaire péritonéale des Rongeurs. in *Comptes Rendus des Séances de la Société de Biologie et de ses Filiales* 354–357 (1917).

[6] Gabrielli, S. *et al.* Molecular and phylogenetic analysis of the filarial nematode *Micipsella numidica* from the hare Lepus europaeus in Italy. 503–507 (2017) 10.1017/S0022149X15000498. 10.1017/S0022149X15000498 26123728

[7] Rao MAN (1938). *Micipsella indica* n. sp. Indian J. Vet. Sci. Anim. Husb..

[8] Lyons ET, Hansen MF (1961). Observations on *Micipsella brevicauda* n. sp. (Nematoda: Filarioidea) from the Black-Tailed Jack Rabbit, *Lepus californicus melanotis* Mearns, in Southwestern Kansas. Trans. Am. Microsc. Soc..

[9] Hua CJ (2019). Morphology is not a reliable taxonomic tool for the genus Lernaea: Molecular data and experimental infection reveal that *L. cyprinacea* and *L. cruciata* are conspecific. Parasites Vectors.

[10] McNulty SN, Mitreva M, Weil GJ, Fischer PU (2013). Inter and intra-specific diversity of parasites that cause lymphatic filariasis. Infect. Genet. Evol..

[11] Gager Y (2016). The value of molecular vs. morphometric and acoustic information for species identification using sympatric molossid bats. PLoS ONE.

[12] Binkienė R, Chagas CRF, Bernotienė R, Valkiūnas G (2021). Molecular and morphological characterization of three new species of avian Onchocercidae (Nematoda) with emphasis on circulating microfilariae. Parasit. Vectors.

[13] Lefoulon, E., Bain, O., Bourret, J., Junker, K. & Guerrero, R. Shaking the tree : Multi-locus sequence typing usurps current Onchocercid (Filarial Nematode) phylogeny. 1–19 (2015) 10.1371/journal.pntd.0004233. 10.1371/journal.pntd.0004233 PMC4654488 26588229

[14] Rodrigues MS, Morelli KA, Jansen AM (2017). Cytochrome c oxidase subunit 1 gene as a DNA barcode for discriminating Trypanosoma cruzi DTUs and closely related species. Parasit. Vectors.

[15] Blaxter, M. Imagining sisyphus happy: DNA barcoding and the unnamed majority. *Philos. Trans. R. Soc. B Biol. Sci.* **371**, (2016). 10.1098/rstb.2015.0329 PMC4971181 27481781

[16] Laidoudi Y, Medkour H, Levasseur A, Davoust B, Mediannikov O (2020). New molecular data on filaria and its wolbachia from red howler monkeys (*Alouatta macconnelli*) in French Guiana—A preliminary study. Pathogens.

[17] Eamsobhana, P., Lim, P. E. & Yong, H. Sen. Molecular phylogeny of filarial worms (Nematoda: Filarioidea). *Raffles Bull. Zool.* 99–103 (2013).

[18] Graber M (1972). Filaire de la cavité péritonéale et de l ’ appareil circulatoire de lièvres d ’ Europe, d ’ Asie et d ’ Afrique. Ann. Parasitol..

[19] Godfroid, J. Brucellosis. in *Infectious diseases of wild mammals and birds in Europe* (eds. Gavier-Widén, Duff, J. P. & Meredith, A.) 318–328 (Oxford, UK: Wiley-Blackwell, 2012). 10.1002/9781118342442.ch24.

[20] Eberhard ML, Orihel TC (1984). *Loaina gen* n. (Filarioidea: Onchocercidae) for the Filariae Parasitic in Rabbits in North America. Proc. Helm. Soc. Wash..

[21] Highby PR (1938). Vectors, transmission, development, and incidence of *Dirofilaria scapiceps* (Leidy, 1886) (Nematoda) from the Snowshoe Hare in Minnesota. J. Parasitol.

[22] John, L. & George, J. Dirofilaria scapiceps from the rabbit (Sylvilagus Floridanus Mearnsi) in Ohio. **58**, 128–130 (1958).

[23] Forrester, D. *Dirofilaria uniformis* and *Dirofilaria scapiceps* (Nematoda : Filarioidea) from Rabbits in Georgia and South Carolina. (2015).

[24] Price, D. L. *Dirofilaria uniformis* sp. n. (Nem_atoda: Filarioidea) from *Sylvilagus floridanus mallurus* (Thomas) in Maryland. *Proc. Helminhol. Soc. Wash.* 15–19 (1957).

[25] Gubanov, M. N. & Fedorov, K. P. *Dirofilaria timidi* n. sp. from *Lepus timidus*. *Tr. gel’mint Lab.* **17**, 47–48 (in Russian) (1966).

[26] Bartlett CM (1983). *Cercopithifilaria leporwus* n. sp. (Nematoda: Filarioidea) from the snowshoe hare - (*Lepus americanus* Erxleben) (Lagomorpha) in Canada. Ann. Parsitol. Hum. Comp.

[27] Hofing, G. L., Ringler, D. H. & Newcomer, C. E. Arthropod and helminth parasites. in *The biology of the laboratory rabbit.* (eds. Manning, P. J., Ringler, D. H. & Newcomer, C. E.) 231–257 (Academic Press, 1994).

[28] Eberhard ML, Telford SR, Spielman A (1991). A Brugia species infecting rabbits in the northeastern United States. J. Parasitol..

[29] Muhire BM, Varsani A, Martin DP (2014). SDT: A virus classification tool based on pairwise sequence alignment and identity calculation. PLoS ONE.

[30] Duarte, M. D. *et al.* The Health and Future of the Six Hare Species in Europe: A Closer Look at the Iberian Hare. in *Lagomorphs* (IntechOpen (in press), 2020).

[31] Abade dos Santos, F. A. *et al.* First description of a herpesvirus infection in genus Lepus. *PLoS One* **15**, e0231795–e0231795 (2020). 10.1371/journal.pone.0231795 PMC7164596 32302375

[32] Carvalho (2020). First cases of myxomatosis in Iberian hares (*Lepus granatensis*) in Portugal. Vet. Rec. Case Reports.

[33] Duarte, M. D. *et al. +Coelho 2: Desenvolvimento e implementação de medidas práticas impulsionadoras da recuperação dos leporídeos silvestres em Portugal*. (2021).

[34] Kalantarian, E. V. Sur la faune des vers parasites des Rongeurs d’Arménie. 18–33 (1924).

[35] Ivashkin, V. M. Helminths of hares in Mongolia. (1954).

[36] Nega A (2014). Review on Nematode Molecular Diagnostics: From Bands to Barcodes..

[37] Stein LD (2003). The genome sequence of *Caenorhabditis briggsae*: A platform for comparative genomics. Plos Biol..

[38] Christoforou, M., Orford, M. & Tsaltas, D. Molecular Diagnostic Tools for Nematodes. in *Nematology* (eds. Shah, M. M. & Mahamood, M.) (IntechOpen, 2017). doi:10.5772/intechopen.69075.

[39] Bogale M, Baniya A, Digennaro P (2020). Nematode identification techniques and recent advances. Plants.

[40] Brooks, D. R. Parasite Systematics in the 21st Century : Opportunities and Obstacles. **95**, 99–107 (2000). 10.1590/s0074-02762000000700018 11142735

[41] Avó, A. P. *et al.* DNA barcoding and morphological identification of benthic nematodes assemblages of estuarine intertidal sediments: Advances in molecular tools for biodiversity assessment. **4**, (2017).

[42] Brilhante, A. F. *et al.* First report of an Onchocercidae worm infecting Psychodopygus carrerai carrerai sandfly, a putative vector of *Leishmania braziliensis* in the Amazon. *Sci. Rep.* 1–9 (2020) 10.1038/s41598-020-72065-9. 10.1038/s41598-020-72065-9 PMC7498610 32943684

[43] Hebert PDN, Cywinska A, Ball SL, DeWaard JR (2003). Biological identifications through DNA barcodes. Proc. R. Soc. B Biol. Sci..

[44] Tavares ES, Baker AJ (2008). Single mitochondrial gene barcodes reliably identify sister-species in diverse clades of birds. BMC Evol. Biol..

[45] Trivedi S, Aloufi AA, Ansari AA, Ghosh SK (2016). Role of DNA barcoding in marine biodiversity assessment and conservation: An update. Saudi J. Biol. Sci..

[46] Oba Y, Ôhira H, Murase Y, Moriyama A, Kumazawa Y (2015). DNA barcoding of Japanese click beetles (Coleoptera, Elateridae). PLoS ONE.

[47] Strüder-Kypke MC, Lynn DH (2010). Comparative analysis of the mitochondrial cytochrome c oxidase subunit I (COI) gene in ciliates (Alveolata, Ciliophora) and evaluation of its suitability as a biodiversity marker. Syst. Biodivers..

[48] Lin X, Stur E, Ekrem T (2015). Exploring genetic divergence in a species-rich insect genus using 2790 DNA barcodes. PLoS ONE.

[49] Chan AHE, Chaisiri K, Morand S, Saralamba N, Thaenkham U (2020). Evaluation and utility of mitochondrial ribosomal genes for molecular systematics of parasitic nematodes. Parasit. Vectors.

[50] Field KG (1988). Molecular phylogeny of the animal kingdom. Science (80-).

[51] Wainright PO, Hinkle G, Sogin ML, Stickel SK (1993). Monophyletic origins of the metazoa: an evolutionary link with fungi. Science (80-)..

[52] Nakacwa R (2013). Nematode 18S rRNA gene is a reliable tool for environmental biosafety assessment of transgenic banana in confined field trials. Transgenic Res..

