# Author Correction: Genetic and morphological identification of filarial worm from Iberian hare in Portugal

**DOI:** 10.1038/s41598-022-24734-0

**Published:** 2022-11-25

**Authors:** F. A. Abade dos Santos, M. D. Duarte, C. L. Carvalho, M. Monteiro, P. Carvalho, P. Mendonça, P. C. L. G. Valente, H. Sheikhnejad, H. Waap, J. Gomes

**Affiliations:** 1 Centre for Interdisciplinary Research in Animal Health (CIISA), Faculdade de Medicina Veterinária, Universidade de Lisboa, Avenida da Universidade Técnica, 1300-477 Lisboa, Portugal; 2 Instituto Nacional de Investigação Agrária E Veterinária (INIAV, I.P.), Quinta Do Marquês, Av. da República, 2780-157 Oeiras, Portugal; 3 Associate Laboratory for Animal and Veterinary Sciences (AL4AnimalS), Vila Real, Portugal; 4 InnovPlantProtect Collaborative Laboratory, Department of Protection of Specific Crops, 7350-478 Elvas, Portugal

Correction to: *Scientific Reports* https://doi.org/10.1038/s41598-022-13354-3, published online 03 June 2022

The original version of this Article contained an error in the Funding section.

“This research was also funded by Fundação para a Ciência e Tecnologia (FCT), Project UIDB/00276/2020 and by the Interdisciplinary Research Centre on Animal Health (CIISA), Faculty of Veterinary Medicine, University of Lisbon (Portugal).”

now reads:

“This research was also funded by Fundação para a Ciência e Tecnologia (FCT), Project UIDB/00276/2020 and LA/P/0059/2020 - AL4AnimalS, and by the Interdisciplinary Research Center on Animal Health (CIISA), Faculty of Veterinary Medicine, University of Lisbon (Portugal).”

The original Article has been corrected.

