# Supplementary material for: Genetic and morphological identification of filarial worm from Iberian hare in Portugal
Source: Sci Rep. 2022 Jun 3;12:9310. doi: 10.1038/s41598-022-13354-3 (PMC9166702; doi:10.1038/s41598-022-13354-3)
Supplement: Supplementary file 1 — Supplementary Information. [file 41598_2022_13354_MOESM1_ESM.docx]

| Table S1 – Primers used in the study | | | | |
| --- | --- | --- | --- | --- |
| **Gene** | Designation | Sequence (5'-3') | Product size (bp) | Reference |
| ***12S rDNA*** | 12SdegF2/ | ATTACYTATTYTTAGTTTA | ~600 | Lefoulon et al., 2015 ^1^ |
|  | 12SnemR2* | CTACCATACTACAACTTACGC |  |  |
|  | 12SF/ | GTTCCAGAATAATCGGCTA | 450 |  |
|  | 12SdegR | ATTGACGGATGRTTTGTACC |  |  |
| ***coxI*** | FCo1extdF1 | TATAATTCTGTTYTDACTA | ~970 |  |
|  | FCo1extdR1* | ATGAAAATGAGCYACWACATAA |  |  |
|  | COIintF/ | TGA TTG GTG GTT TTG GTA A | 650 |  |
|  | COIintR | ATA AGT ACG AGT ATC AAT ATC |  |  |
| ***myoHC*** | fil_myoHCp_F1 | GCATCARGAAGAAATTAATCG | ~1060 |  |
|  | fil_myoHCp_R1 | GCTTCAATTTCYTCCTCCAT |  |  |
|  | fil_myoHCpN_F2 | GAYGATCTTGAGGACAAYYT | ~785 |  |
|  | fil_myoHCpN_R2* | TCTTCAATYTGTTTBCCRAG |  |  |
| ***hsp70*** | FHsp70_dF3 | TCRGATTTCTTYTCTGGYA | ~790 |  |
|  | FHsp70_dR1 | GTYTGYTTCATATTGAAYGC |  |  |
|  | FHsp70_dF1 | CAGCTATYCTYTCTGGTGAT | ~610 |  |
|  | FHsp70_dR1* | GTYTGYTTCATATTGAAYGC |  |  |
| ***Rbp1*** | RNApolyLSp_dF1 | ACTGCAAAYACWGCWATTTA | ~640 |  |
|  | RNApolyLSp_dR1 | ACRTGATTCATTTCRCGTTC |  |  |
| ***18S rDNA*** | F18ScF1 | ACCGCCCTAGTTCTGACCGTAAA | ~740 |  |
|  | F18ScR1 | GGTTCAAGCCACTGCGATTAAAGC |  |  |

^1^ Lefoulon, E., Bain, O., Bourret, J., Junker, K. & Guerrero, R. Shaking the Tree : Multi-locus Sequence Typing Usurps Current Onchocercid ( Filarial Nematode) Phylogeny. 1–19 (2015) doi:10.1371/journal.pntd.0004233.

|  | **Table S2 -** Genbank access numbers for the sequences used in this study. | | | | | | | |
| --- | --- | --- | --- | --- | --- | --- | --- | --- |
| Species | | **Subfamily** | ***rbp1*** | ***hsp70*** | ***MyoHC*** | ***coxI*** | ***12S*** | ***18S*** |
| *Acanthocheilonema viteae* | | Onchocercinae | KP760264 | KP760411 | KP760213 | KP760169 | KP760315 | KP760117 |
| *Aproctella alessandroi* | | Spendidofilariinae | KP760265 | KP760412 | KP760214 | FR823335 | FR827905 | KP760118 |
| *Breinlia jittapalapongi* | | Onchocercinae | KP760266 | KP760413 | KP760215 | KP760170 | KP760316 | KP760119 |
| *Brugia malayi* | | Onchocercinae | KP760267 | KP760414 | KP760216 | KP760171 | KP760317 | KP760120 |
| *Brugia timori* | | Onchocercinae | KP760269 | KP760416 | KP760218 | KP760173 | KP760319 | KP760122 |
| *Cercopithifilaria bainae* | | Onchocercinae | KP760271 | KP760417 | KP760219 | KP760175 | KP760321 | KP760123 |
| *Dipetalonema gracile* | | Onchocercinae | KP760278 | KP760424 | KP760226 | KP760181 |  | KP760131 |
| *Dipetalonema robini* | | Onchocercinae | KP760280 | KP760426 | KP760228 | KP760183 | KP760329 | KP760132 |
| *Dirofilaria immitis* | | [Dirofilariinae](https://www.marinespecies.org/aphia.php?p=taxdetails&id=1425434) | KP760281 | KP760427 | KP760229 | KP760184 | KP760330 | KP760133 |
| *Dirofilaria repens* | | [Dirofilariinae](https://www.marinespecies.org/aphia.php?p=taxdetails&id=1425434) | KP760282 | KP760428 | KP760230 | KP760185 | KP760331 | KP760134 |
| *Filaria latala* | | *Outgroup* |  | KP760429 | KP760231 | KP760186 | KP760332 | KP760135 |
| *Foleyella candezei* | | [Dirofilariinae](https://www.marinespecies.org/aphia.php?p=taxdetails&id=1425434) | KP760283 | KP760430 | KP760232 | KP760187 | FR827906 | KP760136 |
| *Icosiella neglecta* | | Icosiellinae | KP760285 | KP760432 | KP760234 | KP760188 | KP760334 | KP760138 |
| *Litomosoides hamletti* | | Onchocercinae | KP760288 | KP760435 | KP760237 | KP760192 | KP760337 | KP760141 |
| *Litomosoides solarii* | | Onchocercinae | KP760289 | KP760436 | KP760238 | KP760193 | KP760338 | KP760142 |
| *Loa loa* | | [Dirofilariinae](https://www.marinespecies.org/aphia.php?p=taxdetails&id=1425434) | KP760290 | KP760437 | KP760239 | KP760194 | KP760339 | KP760143 |
| *Madathamugadia hiepei* | | Spendidofilariinae | KP760293 | KP760440 | KP760242 | JQ888270 | JQ888289 | KP760146 |
| *Mansonella perforata* | | Onchocercinae | KP760292 | KP760439 | KP760241 | AM749265 | AM779802 | KP760145 |
| *Onchocerca gutturosa* | | Onchocercinae | KP760303 | KP760449 | KP760251 | KP760201 | KP760347 | KP760156 |
| *Onchocerca volvulus* | | Onchocercinae | CBVM020000075 | J04006 | M74066 | AF015193 | AF015193 | ADBW01003330 |
| *Oswaldofilaria chabaudi* | | Oswaldofilariinae | KP760306 | KP760452 | KP760254 | KP760204 | KP760350 | KP760159 |
| *Pelecitus fulicaeatrae* | | [Dirofilariinae](https://www.marinespecies.org/aphia.php?p=taxdetails&id=1425434) | KP760308 | KP760454 | KP760256 | KP760206 | KP760352 | KP760161 |
| *Rumenfilaria andersoni* | | Spendidofilariinae | KP760309 | KP760456 | KP760258 | JQ888273 | JQ888291 | KP760163 |
| *Setaria labiatopapillosa* | | Setariinae | KP760310 | KP760457 | KP760259 | KP760208 | KP760354 | KP760164 |
| *Setaria tundra* | | Setariinae | KP760311 | KP760458 | KP760260 | KP760209 | KP760355 | KP760165 |
| *Wuchereria bancrofti* | | Onchocercinae | ADBV01001653 | ADBV01000792 | ADBV01001797 | JN367461 | JN367461 | AF227234 |
| *Micipsella numidica* | | Splendidofilariinae |  |  |  | KR232089 | KR091069 |  |
| *Dracunculus medinensis* | | *Outgroup* |  |  |  | NC_016019 | NC_016019 | |
| *Chandlerella quiscali* | | Splendidofilariinae |  |  |  | NC_014486 | NC_014486 | |
